# Acute kidney injury in urological conditions

**DOI:** 10.2478/abm-2026-0002

**Published:** 2026-04-30

**Authors:** Kittisak Weerapolchai, Nuttha Lumlertgul, Nattachai Srisawat, Marlies Ostermann

**Affiliations:** Division of Urology, Somdech Phra Pinklao Hospital, Bangkok 10600, Thailand; Division of Nephrology, Excellence Centre for Critical Care Nephrology, Faculty of Medicine, King Chulalongkorn Memorial Hospital, Bangkok 10330, Thailand; Department of Intensive Care, King's College London, Guy's and St Thomas' Hospital, London SE1 7EH, UK

**Keywords:** acute kidney injury, obstruction, obstructive nephropathy, urology

## Abstract

Acute kidney injury (AKI) is a sudden deterioration of kidney function, typically diagnosed by a rise in serum creatinine (SCr) and/or a decrease in urine output. In urological conditions, AKI can result from a variety of factors such as an obstruction of the genitourinary tract, urological procedures, trauma, vascular causes, nephrotoxic drugs, or infection. Imaging plays an integral part in the diagnosis of post-renal AKI. Novel kidney biomarkers may have roles in the diagnosis and prognostication of urology-associated AKI. Finally, management consists of the correction of the underlying cause and supportive care. The prognosis of AKI in urological conditions depends on the underlying cause, the timeliness of intervention, and the patient's baseline kidney health.

Acute kidney injury (AKI) is a heterogeneous syndrome, affecting 10.0%–15.0% of hospitalized patients and more than 50% of patients in intensive care units (ICU) [1, 2]. AKI is diagnosed by the 2012 Kidney Disease: Improving Global Outcomes (KDIGO) criteria when there is an increase in serum creatinine (SCr) of more than 0.3 mg/dL in 48 h or an increase ≥1.5 times from baseline known or presumed to have occurred within the previous 7 d, or urine output <0.5 mL/kg/h for 6 h. AKI is part of acute kidney disease (AKD), which is defined by a rise in SCr, fall in urine output, an estimated glomerular filtration rate (eGFR) <60 mL/min/1.73 m^2^ or structural criteria for kidney damage for <3 months [3] as shown in **Table 1**. Use of both urine output and SCr criteria improves the sensitivity of detection of AKI and refines prognostic estimates of risk for renal replacement therapy (RRT) and mortality. However, there are some limitations of urine output and SCr. For example, urine output can be affected by measurement errors, physiological response, or diuretics. SCr can be confounded by muscle mass, exogenous protein intake, liver disease, nutritional status, sepsis, fluid resuscitation and conditions which affect tubular SCr secretion [3] **(Figure 1)**. AKI and AKD are associated with short-term and long-term complications, including risks of chronic kidney disease (CKD), end-stage kidney disease (ESKD), and other non-kidney complications [3]. In this narrative review, we aim to discuss the epidemiology, specific causes, diagnostic workup, and treatment of AKI in urology settings with a focus on adult patients.

**Table 1.** KDIGO criteria for kidney disease

**General concept**		
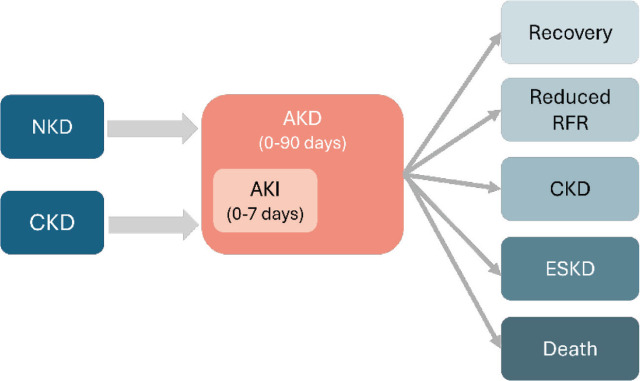		

	**Definition**	**Duration**

**NKD**	**GFR > 60 mL/min/1.73 m^2^**	-
**AKI**	**AKI stage 1:** SCr rise 1.5–1.9 times from baseline in 7 d OR SCr rise by ≥0.3 mg/dL (26.5 μmol/L) in <48 h AND/OR urine output <0.5 mL/kg/h for 6–12 h **AKI stage 2:** SCr rise 2–2.9 times from baseline in 7 d AND/OR urine output <0.5 mL/kg/h for >12 h **AKI stage 3:** SCr rise >3.0 times baseline OR SCr increase to ≥4.0 mg/dL (>353.6 μmol/L) OR decrease in eGFR to <35 mL/min/1.73 m^2^ in patients <18 years OR urine output <0.3 mL/kg/h for ≥24 h OR anuria for >12 h OR initiation of RRT independent of SCr	<7 d
**AKD**	Presence of AKI OR eGFR <60 mL/min/1.73 m^2^ or OR decrease in eGFR by ≥35% OR SCr rise by 50% AND/OR structural markers of kidney damage	0–90 d
**CKD**	eGFR <60 mL/min/1.73 m^2^ AND/OR structural markers of kidney damage	>90 d

AKD, acute kidney disease; AKI, acute kidney injury; CKD, chronic kidney disease; eGFR, estimated glomerular filtration rate; ESKD, end-stage kidney disease; KDIGO, kidney disease improving global outcomes; NKD, no kidney disease; RRT, renal replacement therapy; SCr, serum creatinine concentration.

**Figure 1.**
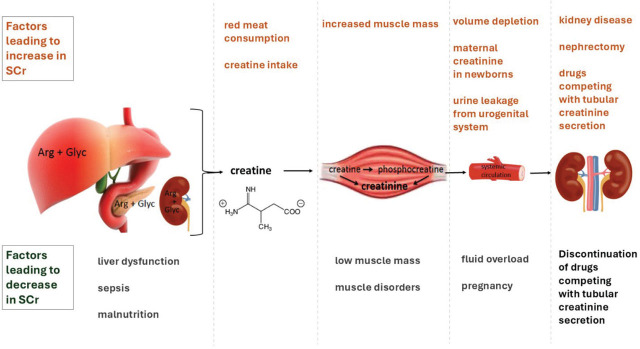
Factors that affect SCr concentrations. SCr, serum creatinine.

## Search strategy and selection criteria

Data for this review were identified by searches of PubMed and MEDLINE for original research papers, narrative reviews, systematic reviews, and meta-analyses, published until May 31, 2025. Search terms used were: “acute kidney injury,” “acute renal failure,” “renal replacement therapy,” “urology” and “obstruction.” We gave preference to citations from the last 5 years published in English. However, we included older papers when studies from the last 5 years were not available. Additional references were selected from relevant articles and textbook chapters.

## Epidemiology of AKI in urology settings

Urological conditions contribute to 7.0%–10.0% of hospitalized AKI [1, 4,5,6] and 1.0%–3.0% of AKI in critically ill adults [2, 7]. In patients admitted to the urology department, AKI occurred in 6.7% of all admissions [8]. However, the incidence of urology-related AKI in outpatient settings and AKD is unclear. In children, 1% of all AKI causes were attributed to urinary obstruction [9]. The prevalence of AKI related to urological procedures varied from 1% in minor procedures, e.g., extracorporeal shockwave lithotripsy (ESWL) or percutaneous nephrolithotomy (PCNL), and up to 65% post-nephrectomy. Patients who underwent emergent procedures were more likely to have AKI than those who had elective procedures [8]. The majority of the patients had AKI stage 1 (89.7%) and experienced a single episode of AKI (88.7%) [8]. In non-elective settings, AKI occurred most frequently from urinary retention, followed by infection and stones [1]. The mortality rate of AKI in patients in the urology department was lower compared to other departments (6.4%) [5].

## Specific causes of AKI in urologic conditions (Figure 2)

### Obstructive nephropathy

Obstructive nephropathy (often referred to as post-renal AKI) results from partial or complete obstruction of urinary flow, leading to impaired kidney function. The etiology of the obstruction may be intrinsic or extrinsic to the urinary tract **(Table 2)**. Post-renal AKI occurs in bilateral obstruction but can develop with a single functioning kidney in cases of lower urinary tract obstruction at any level within the collecting system.

**Figure 2.**
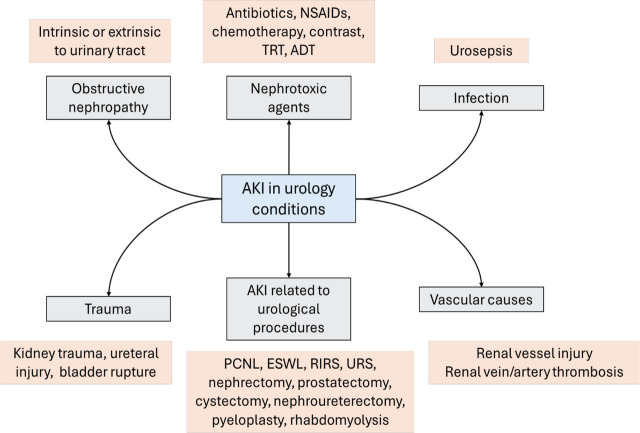
Differential diagnoses of AKI in urological conditions. ADT, androgen deprivation therapy; AKI, acute kidney injury; ESWL; extracorporeal shockwave lithotripsy; NSAIDs, non-steroidal anti-inflammatory drugs; PCNL, percutaneous nephrolithotomy; RIRS, retrograde intrarenal surgery; TRT, testosterone replacement therapy; URS, ureteroscopy.

**Table 2.** Anatomic locations and etiologies of urinary tract obstruction

**Anatomic locations and etiologies of urinary tract obstruction**
Kidney pelvis – Kidney stones Papillary necrosis
Ureter – Bilateral obstruction (patients with CKD, unilateral obstruction with a solitary kidney) Kidney stones, cancer, RPF, severe benign prostatic hypertrophy, neurogenic bladder, stenosis, abscess, ureteral valves, stricture, urinary retention, blood clots, tissues
Posterior to bladder Benign prostatic hypertrophy, cancer, clots
Extrinsic to urinary system – Abdominal and pelvic malignancies, aortic aneurysm, pelvic fractures, atrophic vaginitis, vulvovaginitis, pelvic organ prolapse – RPF – Phimosis Prostate hypertrophy/cancer

CKD, chronic kidney disease; RPF, retroperitoneal fibrosis.

The etiology and the incidence of obstruction vary by age and sex. In children, the most common causes of post-renal AKI are congenital stenosis, vesico-ureteral reflux, and urolithiasis [10, 11]. In young adult males, urinary obstruction is mainly due to urolithiasis, while gynecological surgery or malignancy is the most common cause in young females. In patients >60 years old, urinary obstruction is more frequent in males than in females, commonly due to benign prostate hypertrophy, followed by malignancy. Acute urinary retention, suspected when there is a palpable bladder accompanied by abdominal pain and an inability to pass urine, is common in adults over 65 years and in females during the postpartum period [12, 13]. About 10.0%–12.0% of obstructive AKI cases are caused by urolithiasis. Gravid pregnancy can cause physiological hydronephrosis in up to 90% of women from the ureteral compression by the growing uterus and the effects of progesterone on the tone and peristalsis of the ureters, but rarely causes AKI except in bilateral obstruction [14]. Patients who present with AKI from stones are usually older, have diabetes, CKD, hyperuricemia, have larger or bilateral ureteral stones, or the presence of a solitary kidney [15].

#### Pathophysiology (Figure 3)

There are 3 phases of postrenal obstruction, characterized by distinct hemodynamic changes depending on the duration and type of obstruction (unilateral or bilateral) **(Figure 4)**. In **phase I (1–2 h)**, there is an increase in ureteral pressure and renal blood flow (RBF) due to prostaglandin synthesis and a direct myogenic reflex, resulting in predominant vasodilation of pre-glomerular arterioles. Therefore, glomerular filtration rate (GFR) initially goes up. **Phase II (2**–**5 h)** is characterized by increased ureteral pressure resulting in decreased RBF and GFR. In unilateral obstruction, the predominant site of glomerular vasoconstriction is the afferent arteriole, and in bilateral obstruction, the efferent arteriole vasoconstricts [16]. In **phase III (5**–**24 h),** there is a decrease in ureteral pressure, RBF, and consequently GFR, which are mediated by vasoconstriction of afferent and efferent arterioles from increased production of thromboxane A2 (TXA2) and angiotensin II. The overall glomerular surface area is reduced from mesangial contraction, resulting in a further decrease in glomerular filtration [17]. After 24–48 h, RBF decreases up to 60% and GFR may be reduced by up to 80% [18, 19]. There is a redistribution of intrarenal blood circulation from the cortex to the medulla. Additionally, the lymphatics enhance the movement of interstitial fluid out of the kidney, thereby reducing the intrarenal pressure resulting from obstruction [20]. In unilateral obstruction, tubular pressure and glomerular capillary pressure initially increase, then decline, resulting in reduced GFR in the obstructed kidney. In contrast, tubular pressure progressively increases in bilateral obstruction, and intrarenal blood flow is redistributed in the opposite direction from the medulla to the cortex. In **phases II and III**, the reduction of oxygen delivery to the renal cortex and medulla leads to a subsequent functional loss of the capillary network in the kidney [21]. After 1 week with obstructive nephropathy, microdissection of the renal arterial trees showed a decrease in arteriolar branching; at 8 weeks, with the persistence of the obstruction and reduced blood flow, there was extensive interstitial and tubular damage and tissue atrophy [21]. In a mouse model of unilateral obstruction after 10 weeks, there is progressive development of atubular glomeruli [22]. The glomerulo-tubular junction became atrophic due to autophagy and apoptosis, while the Bowman capsule was simultaneously remodeled to form atubular glomeruli. These are inflamed glomeruli with dysfunctional or non-functioning tubules attached [22].

**Figure 3.**
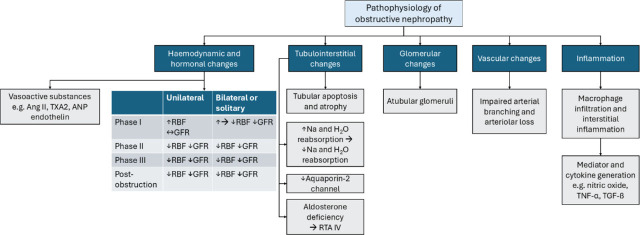
Functional changes during obstruction. Ang II, angiotensin II; ANP, atrial natriuretic peptide; GFR, glomerular filtration rate; NO, nitric oxide; PGE2, prostaglandin E2; Ptubule, tubular hydrolic pressure; Rafferent, afferent arteriolar resistance; RBF, renal blood flow; Refferent, efferent arteriolar resistance; RTA, renal tubular acidosis; TNF-α, tumour necrosis factor α; TXA2, thromboxane A2.

**Figure 4.**
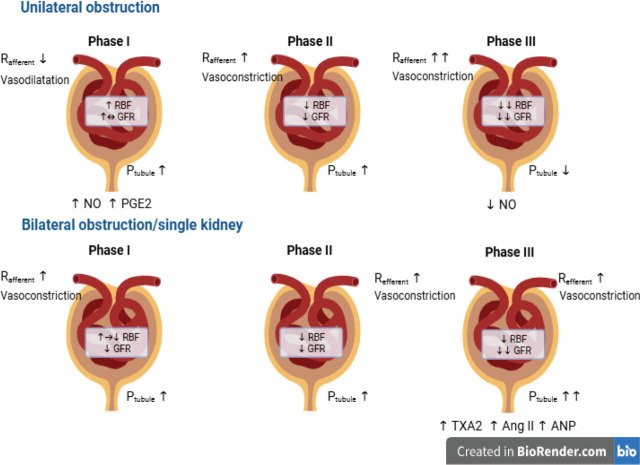
Pathophysiology of obstructive nephropathy. Ang II, angiotensin II; ANP, atrial natriuretic peptide; GFR, glomerular filtration rate; Na, sodium; RBF, renal blood flow; RTA, renal tubular acidosis; TNF-α, tumor necrosis factor α; TGF-β, transforming growth factor β; TXA2, thromboxane A2.

There is an early increase in proximal tubule Na and H2O reabsorption, followed by a reduction in Na and H2O reabsorption within 24 h. Increased Na and Cl delivery to the macula densa stimulates adenosine secretion and causes vasoconstriction of afferent arterioles. There is a downregulation of the aquaporin-2 channel, which persists for up to 7 d after obstruction, leading to post-obstructive polyuria.

Renal acidification is reduced by aldosterone deficiency from hyperreninemia from interstitial damage, leading to the inability to excrete ammonium, potassium, and hydrogen, resulting in a distal renal tubular acidosis (RTA) with hyperkalemia (type IV) [23].

#### Obstructive nephropathy in kidney transplant recipients

Obstruction accounted for approximately 10% of AKI in the early post-transplant period [24]. Compared with acute rejection, obstruction was not associated with a decline in eGFR at 1 year [25]. Causes of urinary tract obstruction in transplant recipients include technical complications of transplant surgery, such as ureteral strictures, extrinsic compression by lymphoceles, hematoma, seroma, urinoma from urine leak at the vesicoureteral anastomosis site, bladder dysfunction due to neurogenic bladder in diabetic patients, or a small non-compliant bladder in previously anuric patients.

#### Non-dilated obstructive uropathy

Non-dilated obstructive uropathy (NDOU) is a post-renal obstructive AKI in the absence of ureteral dilation visible to diagnostic imaging such as ultrasound (US), computed tomography (CT), or intravenous pyelography (IVP) [26]. The reported incidence is about 4.0%–5.0% among post-renal AKI but may be higher due to possible underdiagnosis from false negative rates of the imaging techniques. NDOU is most prevalent in males and typically occurs in the fifth and sixth decades of life. The most common causes of extrinsic compression are retroperitoneal fibrosis (RPF) and malignancy. The most frequently associated malignancies with NDOU are metastatic lesions due to prostate cancer, bladder cancer, lymphoma, colorectal cancer, breast cancer, or, rarely, pancreatic or uterine cancer. The lack of dilation of the calyceal-pyelocalyceal system could be due to several mechanisms. First, there might be an incomplete obstruction, characterized by the fibrous infiltration of only the muscular layer, but not the mucosa or the ureteral lumen, leading to a “functional” obstruction due to decreased peristaltic activity of the ureter. Second, in cases of acute, severe bilateral obstruction, glomerular filtration may continue despite complete obstruction for up to 6 h [27]. There is a pyelosinus extravasation of both the lymphatic and venous systems of the kidney, resulting from continuous water reabsorption by the tubules, which helps to decompress the system, leading to the absence of dilation. Next, mucosal edema and cellular debris can cause early and sudden obstruction before dilation can occur. Lastly, a particular morphology of the renal pelvis could be associated with NDOU. A dendritic (ramified) pattern, which features long, slim, and ramified calyces, is more frequently associated with NDOU compared with an ampullary pattern characterized by short calyces, which drain almost directly into the wide renal pelvis. Additional causes of NDOU syndrome are dehydration, sepsis, and hypotension.

NDOU should be suspected in middle-aged men with AKI and a history of abdominal-pelvic malignancy (especially metastatic cancer), an acute onset of severe oliguria/anuria, and very high SCr in the presence of minimal or absent uremic symptoms. Moreover, fever could indicate an NDOU with a superimposed urinary tract infection. Gastrointestinal symptoms, such as constipation and diarrhea, may indicate bowel obstruction or colonic mass as contributing factors to the urinary tract obstruction.

Nevertheless, patients with severe AKI should undergo quick surgical decompression. The definitive diagnosis may be obtained by performing an antegrade or retrograde pyelography. The latter is the only imaging technique capable of demonstrating the site and presence of urinary system obstruction. The choice of 1 diagnostic technique over the other is usually based on the intended treatment: anterograde pyelography is chosen if the intended treatment is percutaneous nephrostomy. In contrast, retrograde pyelography is selected if the intended treatment is the placement of a stent. An alternative diagnostic imaging method is diuretic scintigraphy; however, it is rarely used in an acute setting.

#### Prognosis

The degree of renal recovery depends on the extent and duration of the obstruction, as well as the presence or absence of infection. Prolonged obstruction typically results in slow and only partial recovery. In patients with AKI from obstructive nephropathy, complete kidney recovery at the time of hospital discharge, defined as a SCr less than 27 μmol/L above baseline, occurred in 58% of obstructive AKI patients, more in the non-elective than elective procedures (64.5% vs 44.5%) and in the partial nephrectomy than radical nephrectomy (RN) (57.0% vs 37.0%) [8]. In a multicenter study of 93 children with anuria caused by stones, kidney function fully recovered in 57% and improved in 37.6%, with significant improvement occurring over the first 72 h after intervention in 84% [28]. Up to 15.0%–23.0% of patients who presented with AKI and obstruction required short-term RRT [28], although early relief of obstruction and prompt correction of electrolyte and acid–base imbalances may minimize this risk.

Following a complete unilateral occlusion for 7 d, 100% recovery of renal function was observed in dogs once the outflow was restored. If the obstructive period was prolonged for 14 d, renal recovery following decompression declined to 70% and a further 30% if obstruction persisted for 4 weeks [29]. After 6 weeks of complete unilateral obstruction, the absence of renal recovery was observed despite drainage of the urinary tract [30]. In rat models, a decline in GFR and RBF following 7 d of complete unilateral obstruction was reported and diminished to 40% up to 30 d following urinary drainage [31]. In patients with bilateral malignant ureteral obstruction, female sex, low body mass index (BMI), SCr at presentation, and parenchymal thickness (PT) were predictors of rapid SCr normalization [32]. Concomitant acute pyelonephritis, AKI, and an obstruction duration of >7 d were independently associated with non-recovery.

Mishra et al. [33] recently developed a model to predict renal recovery in patients presenting with bilateral obstructive nephropathy, which is the blockage of the urinary tract that impairs urine flow, resulting from stones. Combined renal cortical width, proteinuria, nadir GFR at days after relief of obstruction, and a positive urine culture were used to develop a 1-year renal deterioration index (RDI). The RDI had a good predictive value (Area under the Receiver Operating Curve (AUROC) of 0.90) for predicting renal functional outcome. Those with an RDI greater than 12 at presentation had a markedly increased risk of having CKD stage 5 at 1 year.

## AKI related to urological procedures

When kidney tissue is removed, there might be a rise in SCr, which is a functional marker. However, this might not represent tubular injury or damage. The current AKI criteria cannot differentiate the loss of GFR by the removal of kidney tissue from true tubular injury. Among elective urological procedures, nephrectomy, nephroureterectomy, cystectomy, and prostatectomy are frequently associated with AKI. The primary mechanism is likely tubular injury, as demonstrated by an increase in damage biomarkers. Risk factors are categorized into 3 groups: preoperative, intraoperative, and postoperative **(Table 3)**.

**Table 3.** Risk factors for AKI during urological procedures

**Pre-operative**	**Intra-operative**	**Post-operative**
Age Smoking Low baseline eGFR Proteinuria Solitary kidney High BMI Receipt of ACEI Hypertension Diabetes Higher uric acid levels Anemia R.E.N.A.L. score (nephrectomy) PADUA score (nephrectomy) Tumour size ASA score Preoperative obstructive uropathy Neoadjuvant chemotherapy	Bilateral PCNL Longer operation time Larger stone volume Intraoperative bleeding Multiple procedural tracts Induced pneumoperitoneum during LPN Intraoperative hypotension WIT Open nephrectomy (compared with LPN and RAPN) High estimated kidney volume loss (>5.5 cm) Console time Retropubic RP (compared with RALRP) Radical cystectomy with nephroureterectomy Clamping time Fluid balance ERAS protocol	Postoperative ureteric obstruction Postoperative urinary tract infection Bleeding Nephrotoxic agents

ACEI, angiotensin-converting enzyme inhibitors; AKI, acute kidney injury; ASA score, American Society of Anesthesiologists Physical Status Classification; BMI, body mass index; eGFR, estimated glomerular filtration rate; ERAS, enhanced recovery after surgery; LPN, laparoscopic partial nephrectomy; PCNL, percutaneous nephrolithotomy; RALRP, robot-assisted radial laparoscopic prostatectomy; RAPN, robot-assisted partial nephrectomy; RP, radical prostatectomy; WIT, warm ischemic time.

### PCNL

PCNL is considered for patients with large stones (>2 cm), staghorn calculi, complex stones, or patients with anatomical abnormalities. The procedure involves inserting a tube through a small incision and then removing the stones via a nephroscope.

Generally, eGFR as calculated by the modified diet in renal disease (MDRD) equation increases after the stone burden is relieved, more so in patients with bilateral kidney stones than in those with single kidney stones [34]. However, AKI can occur in 4.4%–25.0% after PCNL. There are significantly increased levels of kidney injury biomarkers, such as kidney injury molecule-1 (KIM-1), neutrophil gelatinase-associated lipocalin (NGAL) and N-acetyl-β-glucosaminidase (NAG), compared to preoperative values [35]. Independent risk factors for AKI were age, smoking, female sex, high baseline SCr, solitary kidney, BMI >28.5 kg/m^2^, the receipt of preoperative angiotensin-converting enzyme inhibitors (ACEI), hypertension, diabetes, higher uric acid levels, preoperative anemia, bilateral PCNL, longer operation time, larger stone volume, intraoperative bleeding, multiple procedural tracts and postoperative ureteric obstruction [34, 36,37,38,39]. Patients with AKI had a significantly longer hospital and ICU length of stay. At the 3-month follow-up, 92% had recovered completely from AKI, and 8% had developed stage 4 CKD [37].

### ESWL

ESWL is a non-invasive procedure that uses high-energy shock waves to break down kidney stones, allowing them to pass into the urine. It is usually indicated for smaller stones located in the kidney or proximal ureter. ESWL is associated with short-term structural and functional injury, as demonstrated by transient increases in KIM-1, NGAL, and serum cystatin C levels [40, 41]. There is no clear evidence of long-term detrimental effects on the kidneys.

### Retrograde intrarenal surgery and ureteroscopy

Retrograde intrarenal surgery (RIRS) and ureteroscopy (URS) are minimally invasive procedures for the removal of kidney stones. RIRS utilizes a flexible scope to access stones within the kidney, whereas URS employs a semi-rigid scope for stones in the ureter. After RIRS, there is a significant increase in clusterin, Glutathione S-transferase π (GST-π), β2 microglobulin (β2 M), NGAL, and cystatin C, all of which gradually normalize over the first 14 postoperative days [42]. Another study found a 13.3% incidence of AKI after RIRS. Stone size, operative time, postoperative urinary tract infection and diabetes mellitus are significant predictors of AKI [43].

The irrigation procedure during RIRS increases the intrarenal hydrostatic pressure, and therefore it can hurt renal function by reducing the GFR and tubular transport function. Ureteral access sheaths (UASs) are used to facilitate the access of the flexible ureterorenoscopy (FURS) to the renal collecting system during RIRS. Therefore, a smaller diameter of UAS may be associated with increased intrarenal pressure and a higher risk of tubular injury. In a randomized controlled trial of 125 patients undergoing RIRS for kidney stones, patients who were randomized to smaller UASs (9.5/11.5 Fr) were associated with increased urinary KIM-1, NAG, and NGAL levels compared with larger UASs (12/14 Fr) [44]. Similarly, changes in NGAL levels were observed after URS, which disappear over time [45]. Post-URS AKD occurred in 11.3% of patients, with recovery found in 82% patients. CKD, operative time, and postoperative complications were independent predictors of AKD.

### Nephrectomy

Nephrectomy is indicated for various kidney conditions. In patients with small renal masses (<4 cm), current guidelines recommend surgery (either partial nephrectomy or RN) as the standard of care, but thermal ablation (TA) and active surveillance (AS) are acceptable approaches depending on patient comorbidities and preference [46]. For large masses, surgery is the mainstay of treatment. Patients with large kidney stones may require nephrectomy. In addition, nephrectomy in kidney transplant donors warrants discussion regarding long-term kidney function.

RN involves the complete removal of the kidney, perinephric fat, regional lymph nodes, and/or ipsilateral adrenalectomy. In partial nephrectomy or nephron-sparing surgery, only the tumor or diseased part of the kidney is removed. Regarding surgical techniques, nephrectomy can be performed using either an open or a minimally invasive approach, including laparoscopic partial nephrectomy (LPN) or robot-assisted partial nephrectomy (RAPN).

Mechanisms of postoperative AKI include reduced renal perfusion and reperfusion injury, inflammation, intraoperative factors, and nephrotoxic agents. At the same time, compensatory hyperfiltration after nephron loss and AKI leads to a transition to CKD. Urinary NGAL increased significantly after surgery from 20.02 ng/mL to 56.36 ng/mL [47]. In LPN, the induced pneumoperitoneum required for adequate visualization of the intraabdominal cavity is associated with higher risks of AKI from increased intraabdominal pressure and sub-sequent compression of renal parenchyma and its vasculature. The increased intraabdominal pressure can also compress the inferior vena cava (IVC), which could ultimately decrease venous return and increase arterial vascular resistance [48]. In renal cell carcinoma, which involves IVC, an RN with IVC thrombectomy is often performed. After the IVC is clamped, the venous return decreases, which subsequently leads to decreased cardiac output, systemic hypotension, and elevated venous pressure in the contralateral kidney, all of which can contribute to kidney hypoperfusion.

AKI and CKD are highly prevalent after nephrectomy. AKI is reported in 9.0%–65.0% of patients undergoing nephrectomy [49,50,51,52,53,54,55]. Risk factors of postoperative AKI include age, sex, R.E.N.A.L. score, tumor size, BMI, preoperative eGFR and proteinuria, solitary kidney, baseline hypertension, warm ischemic time (WIT), intraoperative hypotension, ASA score, intraoperative urine output, operation time, intraoperative transfusion, and intraoperative hypotension [50, 55,56,57,58,59,60,61,62,63]. Most importantly, RN increases the risk of AKI and CKD compared with PN despite comparable oncologic outcomes [58, 64,65,66]. Regarding operative techniques, open nephrectomy is more associated with AKI and CKD than minimally invasive LPN and RAPN. In RAPN, high estimated kidney volume loss (>5.5 cm) and long WIT (>25 min) increased the risks of AKI [67, 68]. WIT also correlated with AKI staging [69].

Interestingly, the use of tissue adhesive for tumor bed closure increased the risk of AKI compared with suture [31]. The use of off-clamp RAPN marginally increased blood loss without providing any benefit to renal function [70]. Intraoperative propofol did not reduce the incidence of AKI compared with desflurane [71]. Use of kidney-adjusted Enhanced Recovery After Surgery (ERAS) protocol did not show a protective effect on the development of AKI, but might be associated with less CKD upstaging long-term [72].

Several models have been developed to predict the risk of AKI after nephrectomy. Kim et al. [73] developed a model using the size of the parenchymal mass removed, male sex, diabetes mellitus, warm ischemia time of ≥25 min, and an immediate postoperative neutrophil count of ≥12,000/μL. The accuracy of the scoring system was 0.827 (95% CI: 0.789–0.865) in predicting AKI. Another model was developed using machine learning techniques to predict postoperative SCr by incorporating total anesthesia time, type of surgery (robotic vs open), preoperative SCr, age, sex, height, tumor size, and 5 features of the Preoperative Aspects and Dimensions Used for an Anatomical Classification (PADUA) score, resulting in 85.5% sensitivity and 99.7% specificity [74]. This could facilitate the integration of real-time risk prediction models into electronic health systems.

Most AKI episodes were stage 1, with <5% of the patients reaching AKI stage 3 [75]. Postoperative AKI was associated with an increased rate of in-hospital mortality, transfusion, prolonged length of stay, and higher hospital costs [76].

New-onset CKD and progression of CKD after nephrectomy are reported in 19%–38% and 24.5%–32%, respectively [52, 77, 78]. Risk factors for CKD development include the percentage of visceral adipose tissue, age, BMI, longer operative time, diameter classification by Diameter-Axial-Polar score, DM, preoperative albuminuria, preoperative eGFR, percentage of kidney preserved, WIT, and RN [53, 64, 78,79,80,81]. Importantly, AKI is a significant predictor for CKD, especially in higher staging and longer duration of AKI [75, 82, 83]. RAPN was shown to have a more favorable outcome than open partial nephrectomy (OPN) in terms of preserving long-term renal function [84].

Martini et al. [85] developed a nomogram to predict eGFR reduction (≥25% from baseline) between 3 months and 15 months after RAPN. The model ultimately included age, sex, Charlson Comorbidity Index (CCI), baseline eGFR, RENAL nephrometry score, AKI, and AKI on top CKD. The model has since been externally validated in patients undergoing OPN, LPN, and RAPN with high accuracy [51, 86].

Another nomogram was developed and internally validated for RAPN to predict 3-year CKD progression. The model included baseline eGFR, solitary kidney status, multiple lesions, RENAL nephrometry score, clamping technique, and AKI. The model accurately predicted 3-year CKD-upstaging (C-index 84%) [87].

The overall rate of ESKD after nephrectomy is low (0.4%–2.8%). A 2017 meta-analysis showed that the final eGFR decreased by 10.5 mL/min/1.73 m^2^ in RN compared with partial nephrectomy (PN), indicating a higher risk of CKD and ESKD. However, the cardiovascular benefits of PN over RN have not been consistently shown [65, 88]. In living kidney donor nephrectomy, the pooled overall ESKD was low (0.4%). However, the data demonstrated an increased risk of CKD (HR: 13.59, 95% CI: 9.42–19.61). The risk of ESKD did not reach statistical significance (HR: 3.29, 95% CI: 0.94–11.51) [89]. Obesity (BMI >30 kg/m^2^), male sex, and donor age are associated with inferior outcomes [90].

### Nephroureterectomy

Extirpative surgery with removal of the kidney, entire ureter, and bladder cuff, or radical nephroureterectomy (RNU), is the treatment of choice for non-metastatic high-risk upper tract urothelial carcinoma. In a retrospective cohort study of 89 patients, AKI occurred on postoperative day 1 in 50.6%. Preoperative eGFR was an independent predictor of AKI. Postoperative AKI, lack of ipsilateral preoperative hydronephrosis, preoperative eGFR, and antiplatelet therapy were found to be independent predictors of an eGFR reduction higher than 40% when followed up at a median of 15 months [91].

### Prostatectomy

For prostate cancer, a range of surgical options are available, including open radical prostatectomy (RP) and minimally invasive, laparoscopic or robot-assisted prostatectomy. Early and late AKI developed in 46.9% and 3.9% patients, respectively [92]. Most AKI cases were stage 1. Risks of AKI are related to hypertension, preoperative obstructive uropathy, older age, pre-existing CKD, console time, and postoperative bleeding [8]. One study compared the risk of AKI in 2 dominant surgical approaches using propensity matching. The risk of AKI was significantly higher with retropubic RP than with robot-assisted laparoscopic RP [93]. This might be explained by higher blood loss and more intraoperative blood transfusions in the retropubic RP. Other AKI risk factors included a decrease in cardiac output and kidney perfusion, decreased oxygen delivery, and increased oxidative stress. The eGFR declined 12 months after surgery in patients with early AKI by 6.8 mL/min [92].

### Cystectomy

Bladder cancer accounts for 90%–95% of urothelial carcinomas and can present as non-invasive to aggressive or advanced stage disease. Radical cystectomy includes the complete removal of the bladder and bilateral lymphadenectomy and requires some form of urinary diversion, namely, ileal conduit, continent cutaneous, or orthotopic neobladder. AKI in this setting is due to not only the traumatic and hemodynamic effects but also the addition of urinary diversion [94]. Acutely, urinary diversion can cause AKI through ureteroileal anasto-mosis obstruction and pyelonephritis [95, 96]. When radical cystectomy is concomitantly done with nephroureterectomy for upper genitourinary urothelial carcinomas, the risk of AKI is significantly higher, possibly due to longer surgery times, higher rates of blood loss, hypotension, hypothermia, and associated loss of kidney parenchyma [95].

Early postoperative AKI occurred in 11.0%–33.0%, most of which were stage 1 [60, 97, 98]. Increased risks were reported in surgery >400 min, male, hypertension, neoadjuvant chemotherapy, BMI >25 kg/m^2^, Charleson Comorbidity Index ≥2, intraoperative hypotension (<60 mmHg), intraoperative blood loss, preoperative SCr values, BMI, clamping time (>210 min), fluid balance, performing nephroureterectomy with cystectomy and the development of high-grade complications [95, 99]. Intraoperative administration of crystalloids was a pro-tective factor [100]. It is unclear whether urinary diversion type (ileal conduit vs neobladder) affects AKI risk [101, 102]. Use of an ERAS protocol following radical cystectomy was associated with a higher risk of postoperative AKI in patients who had baseline CKD, which could be related to the restricted perioperative fluid management and the use of non-steroidal anti-inflammatory drugs (NSAIDs) [103]. AKD and CKD developed in 54% and 32.5%, respectively [94, 102]. AKI, age, preoperative eGFR, RENAL score, ischemia time >30 min, intraoperative blood loss >300 mL, and urinary tract complications were significant predictors of AKD and CKD [103].

### Pyeloplasty

In infants with bilateral ureteropelvic junction obstruction (UPJO) who underwent pyeloplasty, AKI occurred more frequently when the intervention was performed on the poorer side first (64%) than the better functioning side (33%) [104]. In contrast, kidney function trajectories varied widely in adult patients with solitary kidneys who underwent pyeloplasty, with static, improving, and decreasing trajectories observed in 63%, 27%, and 10%, respectively. There was a significant increase in renal parenchymal volume (RPV) and PT. A functional success rate of 90.4% was achieved. Patient's age, PT ≤ 0.75 cm and higher, early postoperative AKI staging were associated with increased risk of eGFR decline [105].

#### Rhabdomyolysis

In major urological surgery, rhabdomyolysis is a rare complication (0.03%) from immobility after radical cystectomy and urethroplasty. Male sex, younger age, lower ASA score, prolonged operative time, high BMI, elevated preoperative SCr, and estimated blood loss were identified as possible risk factors. In contrast, a higher intraoperative administered volume was found to be a possible protective factor [106].

#### Nephrotoxic drugs

Urology patients are at risk of nephrotoxin-associated AKI. For example, they may receive preoperative antibiotics such as gentamicin or ciprofloxacin or contrast media during investigation for obstruction. Postoperatively, they may receive NSAIDs for pain control. In patients with urologic cancer who receive standard chemotherapy, targeted therapies or immunotherapy may increase risks of AKI. Patients may receive contrast before or during urological procedures, which may cause contrast-associated AKI (CA-AKI). However, the exact incidence of CA-AKI is unknown.

Among men with hypogonadism, testosterone replacement therapy (TRT) is often prescribed. The impact of TRT on renal function remains controversial. In 1 observational study, long-term TRT was associated with lower serum uric acid and improved eGFR [107]. Among hypogonadal men undergoing urologic surgery, including radical cystectomy and radical nephrectomy, TRT was not associated with postoperative AKI [108, 109]. In contrast, hypogonadal men with underlying cardiovascular risk factors or pre-existing cardiovascular disease who receive TRT may be at increased risk of AKI after starting therapy [110].

Androgen deprivation therapy (ADT) is the mainstay of treatment for patients with advanced prostate cancer. While this therapy has been traditionally reserved for patients with advanced diseases, ADT is increasingly being used in patients with less severe forms, such as in patients with biochemical relapses. ADT may antagonize the vasodilating effects of testosterone on renal vessels while also creating an estrogen deficiency, which can negatively affect renal tubular function [111]. In a UK cohort of 10,250 patients, the current use of any ADT was associated with an increased risk of AKI when compared with never users. The eGFR decreased with the duration of ADT treatment and improved after treatment discontinuation [112].

#### Infections

Urosepsis is sepsis caused by infections of the urinary tract, including cystitis or pyelonephritis. In urosepsis, about one-third developed severe AKI. Prostatitis can cause AKI through sepsis or as a consequence of an underlying condition such as an inflamed prostate or benign prostate hyperplasia (BPH). Age, sepsis, lactate values, and disseminated intravascular coagulation were significantly associated with severe AKI development. Performance status and severe AKI increased 30-d mortality risks [113].

#### Trauma

Blunt or penetrating trauma to the kidneys can cause AKI [114]. Independent predictors included increasing age, male sex, and blood transfusions [115]. Renal infarction may complicate blunt abdominal trauma. Kidney trauma, ureteral injury, or bladder rupture may occur following trauma or procedures such as transurethral resection of bladder tumor (TUR-BT) or radiation therapy. Following bladder rupture or ureteral leak, patients may present with abdominal distension due to ascites. Peritoneal fluid urea, creatinine, and potassium levels are higher than those in serum. This is so-called “pseudo-renal failure [116].”

#### Vascular causes

In urologic cancer, patients may have renal vein thrombosis, which results in AKI. Patients may present with anuria, gross hematuria, or flank pain. Renal artery thrombosis is a rare complication following nephrectomy, kidney transplant, or may occur spontaneously [117, 118]. Additionally, renal vessel injury may complicate urological interventions.

## Diagnostic work-up

### Clinical presentation

In upper urinary tract obstruction, patients may present with flank pain from renal colic or signs of superimposed infection. In bladder outlet obstruction, patients may present with acute urinary retention and a palpable and tender suprapubic mass. Digital rectal or vaginal assessment may reveal causes. Anuria is usually observed in cases of bladder outlet obstruction, trigonal invasion by pelvic malignancy, bilateral obstruction, or obstruction in a solitary kidney. Urine output is often decreased or absent due to a functional or structural impediment to urinary flow [119]. AKI is confirmed by SCr and/or urine output according to the KDIGO criteria.

### Imaging

Conventional ultrasonography plays a crucial role in diagnosing urinary obstruction [120]. Ultrasonography can exclude obstruction in the absence of hydronephrosis with low clinical suspicion, with a negative predictive value of 98%. However, the presence of a urinary tract dilation does not necessarily indicate the presence of an obstruction, thus reducing its specificity (<70%). Several conditions may lead to a slight dilatation of the pelvicocalyceal system, such as hyperhydration, diuretic use, pregnancy, mega-calycosis, or some anatomical abnormalities such as extra-renal pelvis and para-pelvic cysts. Ultrasonography can help visualize clots, benign prostate hyperplasia, or a distended bladder. Color Doppler can partially overcome the low specificity of ultrasonography by visualizing the ureteral jet and measuring renal resistive indexes (RRI). The presence of a ureteral jet could demonstrate the function and patency of the ureter. RRIs are significantly higher in kidneys with obstruction. A twinkling artifact is a color Doppler phenomenon that may facilitate the detection of nephrolithiasis. CT or magnetic resonance imaging (MRI) may provide more detailed information regarding the location of the obstruction and its causes.

### Roles of biomarkers in diagnosis and management

Serum cystatin C and SCr levels can be a clue in the differential diagnosis of a post-renal cause [121,122,123]. Tsuda et al. [121] found that the increment in serum cystatin C and β2M was much smaller in the bilateral ureteral obstruction (BUO) than in the bilateral nephrectomy (BNX) rats, whereas increases in SCr were similar in the BUO and BNX groups. The ratio of SCr to cystatin C and serum β2M in the post-renal group was higher than in the nephrectomy group (20.6 and 8.3 vs 18.5 and 5.3, respectively). Similarly, Fujisawa et al. [122] reported no or only a minimal increase in serum cystatin C in 3 patients with post-renal obstruction, compared with a markedly increased SCr. These studies suggest that some low-molecular-weight proteins, such as cystatin C, are freely filtered by the glomerulus, followed by tubular reabsorption, even in cases of obstructive uropathy. Cystatin C is subsequently reabsorbed and almost entirely catabolized by proximal tubules without reentering the bloodstream. In contrast, filtered creatinine would return to the bloodstream via tubular back leak, thereby contributing to increased SCr [124].

In urinary tract obstruction, kidney damage biomarker levels, such as urine NGAL, serum NGAL, and urine KIM-1, were elevated in a more sensitive and timely manner than SCr and decreased following surgical intervention to relieve the obstruction. NGAL levels were acutely reduced by 14% within 2 h and by 78% within 6 months [125,126,127]. Of note, urine NGAL showed a moderate correlation with leukocyturia and should be interpreted accordingly [126]. Urine NGAL and cystatin C may predict AKI after nephrectomy and precede the rise in SCr from 3 h up to 24 h [49, 128, 129]. Other biomarkers, such as vanin-1 levels and NAG, were sig-nificantly higher in hydronephrosis [130]. For long-term outcomes, urine KIM-1 and urine liver-type fatty acid binding protein (L-FABP) levels were shown to predict 1-year kidney function deterioration [131].

Another alternative technique is the measurement of kidney tissue oxygenation (μHbO2) and microcirculation by reflectance spectrophotometry and laser Doppler flowmetry (O2C™, Lea, Germany), which was shown to correlate with postoperative AKI [132]. However, these techniques have only been applied in research settings.

### Kidney pathology

Kidney biopsy is rarely done in suspected post-renal cases. Glomerular tuft shrinking, tubular dilatation, and flattened epithelium have been observed in the initial phase of post-renal AKI [133]. Moreover, there might be an expression of Tam–Horsfall protein (THP) in some glomeruli, which suggests urine backflow [134].

## Treatment

The treatment of AKI can be categorized into non-dialytic and dialytic measures. For non-dialytic measures, the KDIGO AKI care recommendations should be followed i.e. the optimization of volume status and hemodynamics, avoidance of nephrotoxic agents, including contrast agents if possible, avoidance of hyperglycemia and monitoring of urine output and SCr. RRT is indicated for patients with complications of AKI that are refractory to conservative management, e.g., hyperkalemia, volume overload, metabolic acidosis, or uremic symptoms.

The treatment of obstructive nephropathy primarily relies on correcting its underlying cause. In urosepsis, appropriate empirical antibiotics should be administered. If a decision of urinary diversion is made, it should be performed as soon as possible. The chosen technique will vary depending on the site of obstruction, patient characteristics, and the treating team's preferences. Upper tract obstruction is usually managed by retrograde diversion using a ureteral double-J stent. If not feasible, diversion with percutaneous nephrostomy should be considered [135]. Urgent urinary diversion is indicated in cases with high-risk infectious complications such as urosepsis and pyonephrosis, as well as in the presence of a single kidney, bilateral upper tract obstruction, previous CKD, or hyperkalemia. In patients with obstructive nephropathy from malignancy, percutaneous intervention resulted in a median survival of 96 d [136]. Another study reported similar survival rates in those who received conservative care compared with an invasive intervention [137]. Following decompression, alpha-1 receptor blockers are recommended because alpha-1 receptors regulate the function of the bladder, urethra, and prostate [138]. In the cases of bladder outlet obstruction, urinary diversion with a urinary catheter should be performed. If urinary catheterization is unsuccessful, a suprapubic catheter should be considered as an alternative.

## Management of post-obstructive diuresis

Post-obstructive diuresis (POD) is characterized by urine volume ≥200 mL for at least 2 consecutive hours following the relief of urinary retention or urine output exceeding 3000 mL within 24 h. Studies suggest that up to 63% of patients experience POD, which lasts approximately 1.8 d after relief of an obstruction [139]. Pathologic POD often lasts ≥48 h and can lead to severe complications, including dehydration and electrolyte imbalances. Mechanisms include osmotic diuresis from urea, reduction in medullary concentrating ability due to vascular washout, down-regulation of the sodium transport mechanism in the thick ascending loop of Henle, and diminished response to antidiuretic hormone. There is an excretion of sodium, potassium, calcium, and magnesium from alterations in the reabsorption of these solutes. Various mediators contribute to POD, such as atrial natriuretic peptide, prostacyclins, prostaglandins, TXA2, and endothelins. In addition, immunological mechanisms can explain POD through macrophage infiltrates, platelet-activating factor (PAF) elevation, and cytokine release. Patients are at higher risk of pathologic POD if the immediate residual urine post-drainage is ≥1500 mL. Other risk factors for POD include lower urinary tract symptoms, diabetes, a history of multiple urethral catheterizations, prostatic hyperplasia, fecal impaction, the use of anticholinergic medications, high sodium bicarbonate, heart failure, central nervous system depression, and urinary retention [140, 141]. There are no correlations between initial SCr, urea, electrolytes, creatinine clearance or presence of hypertension with the severity of diuresis. Longer time from admission to the release of obstruction and absence of POD after the release of obstruction were independent risk factors for CKD. In patients with urinary retention, there is no difference in POD between gradual versus rapid emptying of the bladder [140].

When evaluating patients at risk or who are developing POD, the patient's weight, overall fluid status, urine output, and electrolyte levels should be monitored. Clinicians should also monitor urine volume at 1- or 2-h intervals, as well as urinary osmolality, sodium, and urea levels, regularly. Urinary sodium and potassium levels, as well as urinary osmolality, can help determine whether the patient has urea or salt diuresis. Urea diuresis is often self-limited. However, salt diuresis, especially if the spot urine sodium level is greater than 40 mEq/L, may lead to pathologic polydipsia. A high urine specific gravity indicates the resolution of POD.

In contrast, a low urine specific gravity indicates the body's inability to concentrate the urine, consistent with nephrogenic diabetes insipidus and POD. The type, route, and amount of fluid administration should be tailored to the patient's electrolyte levels and clinical hydration status. Patients with intact cognition should continue oral hydration. The recommendation is to administer no more than 50%–75% of the prior 1 to 2-h urine production. Excess fluid administration should be avoided as it may exacerbate diuresis. Generally, the prognosis of pathological POD is favorable.

## Limitations

This narrative review primarily focuses on studies involving adult populations published in English. AKI was defined according to the criteria used in the individual reports. Future research should investigate the long-term kidney and non-kidney outcomes of AKI within urological conditions using the current criteria of AKI. Additionally, exploring novel biomarkers for their prognostic value and potential to guide interventions in urological patients is essential. Lastly, preventive strategies should be assessed in specific settings, such as AKI associated with urological procedures and kidney transplantation.

## Conclusions

AKI is a frequently observed complication in urological disorders. Obstructive nephropathy, which may result from various etiologies, has a prognosis that relies upon the location of the obstruction and the timeliness of its relief. AKI is a common complication of post-urological procedures and may subsequently progress to CKD. Ultrasonography remains the primary diagnostic modality but may occasionally fail to detect some cases of NDOU. Emerging biomarkers hold potential to enhance the diagnosis of post-renal AKI, such as cystatin C. The cornerstone of treatment for obstructive nephropathy involves prompt relief of the obstruction. Furthermore, all patients experiencing AKI should undergo long-term renal function monitoring, given the established association between AKI, CKD, and ESKD.
